# The Empowering Effect of Embodied Awareness Practice on Body Structural Map and Sensorimotor Activity: The Case of Feldenkrais Method

**DOI:** 10.3390/brainsci11121599

**Published:** 2021-12-01

**Authors:** Davide Crivelli, Massimilla Di Ruocco, Alessandra Balena, Michela Balconi

**Affiliations:** 1 International Research Center for Cognitive Applied Neuroscience (IrcCAN), Catholic University of the Sacred Heart, 20123 Milan, Italy; michela.balconi@unicatt.it; 2 Research Unit in Affective and Social Neuroscience, Department of Psychology, Catholic University of the Sacred Heart, 20123 Milan, Italy; 3 Sesto Senso Feldenkrais Association, 20129 Milan, Italy; massima45@yahoo.it (M.D.R.); alessandra.balena@alice.it (A.B.)

**Keywords:** embodied motor awareness, sensorimotor network, Feldenkrais method, mu band, EEG, action observation, motor imagery, gesture execution

## Abstract

While outcomes of embodied awareness practices in terms of improved posture and flexibility, movement efficiency, and well-being are often reported, systematic investigations of such training effects and of the actual nature, extent, and neurofunctional correlates of learning mechanisms thought to lie at the core of such practices are very limited. The present study focused on the Feldenkrais method (FM), one of the most established embodied awareness practices, and aimed at investigating the neurofunctional outcomes of the somatic learning process at the core of the method by testing the modulations induced by a standardized FM protocol on the complexity of practicers’ body structural map and on the activity of their sensorimotor network during different movement-related tasks (i.e., gestures observation, execution, and imagery). Twenty-five participants were randomly divided into an experimental group—which completed a 28-session FM protocol based on guided group practice—and a control group, and underwent pre-/post-training psychometric and electrophysiological assessment. Data analysis highlighted, at the end of the FM protocol, a significant increase of EEG markers of cortical activation (task-related mu desynchronization) in precentral regions during action observation and in central regions during action execution and imagery. Also, posterior regions of the sensorimotor network showed systematic activation during all the action-related tasks.

## 1. Introduction

Body awareness can be defined as the representational counterpart of the mostly-covert proprioceptive and interoceptive information flow that connotes and pairs with our experiences [1]. When our body is put in action, such form of awareness becomes deeply-linked, though not fully corresponding, even to processing of postural and balance information, muscular and joint stretch, and visceral sensations, up to higher-level representational contents referred to intentions, affordances, and goal-directed action plans. The peculiar and specific form of awareness for the body *in action* is, then, enriched by further pieces of information concerning afferent sensorimotor and visual-spatial data coming from the moving body and the consequent adaptation of the moving body representations. Such level of self-representation can be defined as embodied motor awareness and grounds on the ability to self-attribute an agentive stance—i.e., sensing ourselves as the cause of our actions and their consequences [2,3]. Embodied motor awareness can become a strategic target for empowerment protocols designed to favor the development of self-regulation skills during the interaction between an agent and his/her environment and to strengthen the ability to create integrated representations of the acting self and body, thus promoting self-enhancement and psychophysical well-being [4].

The focus on kinematic chains and on making the sequence of movements conscious is at the core of many embodied awareness practices, often used as methods to promote the mind-body connection in clinical or subclinical conditions, to reach a deeper awareness of the self, or to improve the sensorimotor acuity. As suggested by Jain and colleagues [5], modern methods for promoting greater awareness and/or re-education of movement, by building on long-standing traditions in contemplative practices, center on awareness of the moving body and on processes supporting the execution of motor acts to increase sensorimotor control and psychophysical mastery.

Among them, the Feldenkrais Method (FM) is one of the most established and diffused one, even beyond the field of physiotherapy and motor rehabilitation. It has been originally defined as a somatic education method based on movement and aimed at extending and optimizing the way the body is used to act by working on bodily awareness [6]. The rationale of the method relies on the assumption that increasing awareness of the moving body enables the improvement of movement efficiency during daily activity and decreases dysfunctionality determined by the habitual re-enaction of suboptimal action patterns. To pursue such a goal, the method uses guided exploration practices focused on different sets of movements (both familiar and unfamiliar) in order to train attention, perceptual discrimination, and proficiency in detecting sensorimotor information while moving [7]. The general improvement of the organization of the movement is achieved by exploring and enacting the functional relationship between the kinetic components to be integrated into the action [8]. Besides that, it has been suggested that the implicit learning process subserving such practice also facilitates learning of novel strategies to improve the organization and coordination of body movement, thus developing a richer and more effective spatial and kinesthetic representation of the relationships between body segments [9].

Therefore, among the primary results of regular practice of FM, there is often reported an improvement of posture, balance, and flexibility, as well as the release from muscle tension, the adoption of effective breathing patterns, and a generalized sense of well-being and “centeredness”. Yet, focusing on empirical investigations of the effects of practicing the method, the scenario seems more complex and less defined. Indeed, critical reviews of the available literature exploring the outcomes of protocols using Feldenkrais’ techniques have highlighted that regular practice with the method (mainly by following regular group sessions of practice guided by an expert trainer) can reliably improve balance, especially in older adults, and likely leads to the reduced perceived effort while performing complex actions, improved range of motion and dexterity, decreased pain (even in chronic conditions), increased comfort while moving, and improved perception of the body image understood—according to Gallagher [10]—as the synthesis of perceptual, conceptual and emotional representation of the body [11,12,13]. Nonetheless, the review report also systematically underlines the high risk of bias presented by the available primary studies, as well as, in many cases, poorly reported methods. Such factors undermine the strength of reported results and of their interpretation.

While the inability to identify a consistent set of outcomes can be partly ascribed to the unclear description of methodological issues, to the diversity of target population that have been investigated, and to the heterogeneity of outcome measures that have been used, the seemingly widespread effect of the practice across different population and domains that emerge from the limited available literature is consistent with the notion that the underlying mechanism of action for FM mirrors sensorimotor learning mechanisms leading to increased embodied awareness, more than dysfunction- or condition-specific compensation mechanisms [11,12]. Yet, the actual nature, the extent, as well as the physiological correlates of such sensorimotor learning mechanisms are still the object of investigation, contrarily to neurofunctional correlated of neighboring forms of awareness practices such mindfulness-based meditation [14,15,16].

Investigations of neurofunctional correlates of FM practice or of its outcomes via neuroimaging methods are, indeed, limited, to our best knowledge, to one work by Verrel and colleagues [8], who reported the first exploratory results regarding increased resting-state activity in higher-order motor areas following a brief FM-based practice involving the feet. While those findings suggest the involvement of action-related neural mechanisms in the learning process elicited by at least some forms of FM practice, no complementary evidence for the effect of FM on the activity and responsivity of the sensorimotor network (which is expected to be the primary target of the training) during actual motor tasks are available. Given the peculiar interest of the method on the development of movement dynamics, on motor control, and on awareness of kinematic chains, electroencephalography might be the method of election in the quest for novel findings on neurofunctional mechanisms supporting FM practice and the related somatic-perceptual learning process. The remarkable cognitive and temporal resolution and the low invasivity of such techniques have already favored its application in exercise and movement sciences [17,18,19], and might represent valuable advantages even in research on mechanisms of action underlying movement-based embodied awareness practices. In particular, task-related synchronization and desynchronization of the EEG mu band proved to be an informative marker of activation and responsiveness of the sensorimotor system during movement-related tasks such as action preparation, execution, observation, and imagination [20,21,22]. Monitoring and quantifying the modulations of the mu band during movement-related tasks might then help qualify the neuroplastic changes in sensorimotor activity following a training period of movement-related practices.

Furthermore, the sensitivity of the mu rhythm to different aspects of movement—i.e., action preparation and execution, observation, and imagery [22,23,24,25]—allows for testing the extent of the effects of practicing FM. Namely, the same marker can be used in association with different, though comparable, tasks tapping on observation, imagery, and execution of gestures, and provide information on: (i) the potential influence of training embodied motor awareness on different levels and components of the human ability to encode and implement complex actions; and (ii) the assumption that the somatic-perceptual learning mechanisms supporting the FM are global and can, therefore, lead to a generalized reconfiguration of the way the practicer perceives, encodes, and implements body movements.

Given such premises, this longitudinal study aims at investigating the neurofunctional outcomes of such implicit learning mechanisms by testing the modulations induced by a standardized FM protocol on the activity of the sensorimotor network of practicers during different movement-related tasks (i.e., observation, imagery, and execution of complex gestures). In addition, we will investigate potential training-induced modulations in the complexity of practicers’ body structural map—understood as the conscious visual-spatial representation of one’s own body, i.e., according to the triadic taxonomy of body representation [26], the structural description of the relationships between body parts based on both vision and somatic perceptual information—via a standardized human figure drawing test. Indeed, while the effect of FM-based interventions on effective representations that contribute to the definition of the body image has been previously explored by looking at self-reported acceptance, perception, or contentment of specific problematic body parts [27,28], the actual level of detail of the structural map of practicers’ body seems not to have ever been directly tested.

Since FM aims to promote self-awareness and optimize the ability to regulate the body in action through a somatic–proprioceptive–motor learning, we expected that: (i) participants who completed the FM protocol, compared to a control group, would show an improvement of the definition of their body structural map, as measures via the human figure drawing test, a well-known psychometric tool that has been here adapted to explore the complexity and integrity of the body visual-spatial representation; (ii) participants who completed the FM protocol, compared to a control group, would show heightened responsivity of sensorimotor network during perception, execution, and imagery of complex gestures, as mirrored by electrophysiological markers of the degree of activation of the premotor, motor, and superior parietal structures (i.e., desynchronization of the Mu EEG band).

## 2. Materials and Methods

### 2.1. Sample

Twenty-five (17 females, 8 males) volunteers took part in the study. Inclusion criteria were: age > 18 yo; normal vision and hearing or corrected with aids. Exclusion criteria were: psychiatric or neurological clinical history; previous cranio-encephalic trauma or cerebrovascular events; the presence of overt sensory, motor or cognitive deficits; body image or eating disorders; the presence of metabolic pathologies or other general pathologies that may lead to an alteration of the perception of oneself and of one’s body; concomitant drug therapies based on substances capable of interacting with the functioning of the central nervous system; previous experience of practicing FM.

None of the participants reported ongoing concurrent therapies based on psychoactive drugs, history of neurology, psychiatric, sensory, or motor disturbances, clinical pictures that might alter self- and body perception, and previous experience with FM. The absence of psychological disturbances and body image or eating disorders was checked via individual clinical colloquia performed by an expert psychotherapist. All of the participants had normal or corrected-to-normal hearing and vision.

Participants were allocated to the Experimental (EXP) or Control (CON) group via randomization stratified by age, so to minimize the potential confounding effect of such factors. Between-group statistical comparisons (independent-groups t-tests, chi-square test) confirmed that groups did not significantly differ in terms of demographics and gender distribution. Table 1 reports comparison data for the two groups, the global sample socio-demographic profile, and the outcomes of between-group statistical comparisons. During the study, six participants—three in each group—dropped out due to personal issues that prevented them from taking part in the post-training assessment (three moved to another city, three could not take part in the post-training evaluation due to personal or health-related reasons).

All participants signed and approved a written consent to participate in the study. They were informed on the overall structure of the study, on the characteristics of the assessment procedures, and on the amount of commitment and time that would be requested if they would have decided to take part in the study. The study and relative procedures followed the principles of the Declaration of Helsinki and were reviewed and approved by the Ethics Committee of the Department of Psychology of the Catholic University of the Sacred Heart.

### 2.2. Procedure

The experimental design was a two-branch longitudinal study including a control and an experimental treatment group, with two main assessment steps to test intervention-related effects on electrophysiological activity of sensorimotor structures and on participants’ body structural map. Specifically, participants underwent psychometric and electrophysiological assessments at the beginning and at the end of the intervention. The experimental treatment was based on weekly group sessions of FM practice, while control participants were put on a waitlist.

#### 2.2.1. Training Procedure

The experimental group has completed a protocol based on FM, including 28 weekly sessions of practice guided by an expert FM instructor. Having to comply with the necessary precautions related to the outburst of COVID-19 pandemic, which worsened in the middle of the training phase, the training protocol had to be adapted to the newly-introduced restrictions, and the last 16 sessions had to be delivered online.

The sessions of practice lasted about 1 h and were based on the awareness trough movement (ATM) techniques. Such techniques are typically practiced in a group, guided by an FM instructor. The instructor uses verbal instructions and advice to guide the practicers through different sequences of movements in order to systematically explore the relationship between body parts, as well as between body position, movement, and space. During ATM practice, participants are encouraged to implement the movements by trying to reduce strain and pain, and slowly enough to be attentive to and aware of what they are doing, feeling, and thinking. The correct movement pattern for the body parts that are interested in the sequence of movements is not described in advance in order to promote self-exploration and individual learning.

The control group, instead, was put on a waitlist between the two assessment steps.

#### 2.2.2. Psychometric Assessment

Assessment sessions were held individually, in a neutral and quiet place. Participants were administered a standardized human figure drawing (HFD) test, in which they had to draw a sketch of a person of the same sex, seen from the front and in underwear (this specific instruction was added so to prevent potential discomfort or embarrassment while still trying to obtaining an anatomically-detailed drawing). The test was adapted from the original and implemented to investigate the complexity and integrity of the conscious body structural map.

The original form of the HFD test was devised in 1949 by Machover [29] as a non-verbal projective test used to explore the inner representation of the self and body image [30]. In the version of the test used in the present study, participants were given a blank A4 sheet with the instructions for completing the test and a pencil. The participant was reassured that neither the quality of their sketches nor their drawing skills were relevant to the test and that these would not be assessed in any way. Ad hoc scoring procedures have been here devised in order to quantify the complexity, integrity, and detail of the human figure that was drawn. Namely, we have firstly identified a pre-established list of body parts on both the right and left side of the human figure, and then assigned a score of 1 if the part was present and properly placed and 0 if it was absent or drawn in unnatural location/orientation. Two trained judges independently scored each individual test and in case of inconsistency in their evaluations, a third judge intervened. The total score was computed as the sum of drawn parts.

#### 2.2.3. Electrophysiological Assessment

The psychometric assessment was complemented by task-related electrophysiological recording during experimental tasks tapping on observation, execution, and imagination of complex movements.

The tasks were presented in four blocks separated by brief pauses (E-prime2 software, Psychology Software Tools Inc., Sharpsburg, PA, USA). Each block included 16 trials. Each trial began with the observation (OBS) task, during which participants were asked to carefully observe brief videoclips depicting complex actions involving the upper arms. Then the execution (EXE) and the imagery (IMM) tasks are presented in a randomized order. During EXE, participants had to enact the complex gesture that they have just seen as accurately as possible. During IMM, participants had to imagine performing the complex gesture that they have just seen in the first person, as accurately as possible. EXE and IMM had no time restrictions in order to minimize any potential confound due to time pressure while participants were enacting or imagining the movement sequence. In order to allow for accurate collection of electrophysiological data during EXE and IMM, participants had to press a button at the beginning of those tasks, and then release it when they felt ready to perform or imagine the gesture. This provided a marker for time-locking of task-related EEG tracks.

A fixation cross was briefly presented on the screen at the beginning of each trial. Then, at the beginning of each task, brief instructions were presented so to remind participants of what they had to do to accomplish each specific task (i.e., “Observe”, “Execute”, and “Imagine”). The sequence of complex gestures within blocks was randomized so to prevent potential order biases. The videoclips used in OBS lasted 4 s, on average, the duration of performed/imagined actions during EXE and IMM was, consistently, 4 s. Trials ended with a 2 s blank. A total of 32 complex gestures was devised, video recorded while performed by a male or a female actor, and included in the experimental design (total number of stimuli: 64) after being rated and validated by six independent judges naïve to the Feldenkrais method and two expert FM trainers. Judges were presented with the videos of the 32 complex gestures performed by a male or a female actor and were asked to rate such complex motor acts with regard to their: familiarity, complexity, concreteness, and meaningfulness. Ratings could be expressed on a seven-point Likert scale (from 1 = complete absence of the attributes, to 7 = complete presence of the attribute). They were also asked about the preferred laterality of such actions—i.e., whether they would be more likely performed with the dominant hand, with the non-dominant hand, or whether they can be equally executed with one hand or the other. All of the selected complex gestures pertained to the upper limbs.

EEG data were recorded both during the above-described tasks and at rest, in order to collect baseline reference data. Resting-state data have been collected both with eyes open and closed (a 2-min runs each). EEG was recorded with a DC amplifier (SynAmps system, Compumedics Neuroscan Inc., Charlotte, NC, USA), and then processed offline via Vision Analyzer2 software (Brain Products GmbH, Gilching, Germany). The recording montage was constituted by 15 sintered Ag/AgCl electrodes—Fp1, Fp2, F3, Fz, F4, T7, C3, Cz, C4, T8, P3, Pz, P4, O1, and O2—referenced to linked earlobes and placed according to the 10-10 International System [31]. Electrodes impedance was kept under 5 kΩ and vEOG was recorded in order to keep track of ocular artifacts for subsequent correction and rejection. The sampling rate was set to 1000 Hz, with a 0.01–250 Hz bandpass and a 50 Hz notch input filter.

Both resting-state and task-related data then been filtered offline (IIR 0.5–50 Hz bandpass filter, 48 db/octave) and visually inspected for movement, muscular, and ocular artifacts. EEG segments containing residual artifacts have been manually rejected. Continuous EEG was segmented according to the experimental condition (i.e., resting eyes-open, resting eyes-closed, action observation, action execution, and action imagination). Artifact-free data have then been used to compute condition-specific power-density (PD) spectra by fast Fourier transform (Hamming window, resolution: 0.5 Hz). Average power-density for the mu EEG band have finally been extracted, log-transformed and used to compute neurometric measures relative to the premotor (Fz), motor (Cz), and posterior (Pz) regions of the sensorimotor network. Namely, task-related desynchronization (TRD) in the mu band was computed as a measure of cortical activation by weighting power density during action observation, execution, or imagination overpower density during eyes-open resting state: TRD = PDtask/PDrest. By this calculation, any value lower than 1 would mirror the desynchronization of the mu band (i.e., greater cortical activation) during the task with respect to reference resting-state, while any value higher than 1 would mirror greater synchronization of the mu band (i.e., lower cortical activation) during the task with respect to reference resting-state.

The mu band was selected for the analysis since it is known to be generated by premotor–motor–somatosensory cortex and is, therefore, associated with the activation, responsiveness, and efficiency of the sensorimotor system. Such an EEG rhythm (frequency range: 10–14 Hz) is suppressed (desynchronized) when a person implements or prepares a movement [20,21]. Therefore, desynchronization mirrors the activation of targeted cortical regions. Again it was shown that the mu rhythm can de consistently modulated by the observation and imagination of movements [22].

#### 2.2.4. Statistical Analysis

Pre- and post-training psychometric data and electrophysiological markers of cortical activation in premotor, motor, and posterior areas during action observation, execution, and imagery have been modeled and analyzed by means of linear mixed-effects models. Statistical analyses have been run and checked using PASW (SPSS Inc., Quarry Bay, HK).

As for the analysis of scores at the human figure drawing (HFD) test, Group (EXP vs. CON) and Time (T0 vs. T1) have been included as fixed effects in the statistical model, while the subject was included as a random effect in order to better control for individual differences between participants. As for the analysis of the TRD values for the mu band, independent models were computed for the three tasks (OBS, EXE, and IMM), including Area (premotor, motor, and posterior), Group (EXP and CON), and Time (T0 and T1) as fixed effects, and the subject as a random effect. Moreover, in every analysis, autocorrelations between subsequent assessment data were modeled through a first-order autoregressive covariance matrix in order to accurately investigate potential intervention effects while accounting for time-related confounds. The significance of terms in the fixed effects has been assessed by conditional F-tests; we will then report relative F and p values of the Type III Sum of Squares computations.

Probability values for all pair-wise comparisons used to explore simple effects and simple contrasts have been adjusted according to Bonferroni correction. Furthermore, Cohen’s d values (difference between the group means divided by the pooled standard deviation of the two groups) were calculated and reported as a measure of effect size for statistically significant pair-wise comparisons. Effect sizes have been deemed as small when ≥0.2, medium when ≥0.5, and large when ≥0.8, in agreement with Cohen’s norms [32].

## 3. Results

The analysis of the scores at the HFD test did not reveal significant main or interaction effects (EXP: EM_t0_ = 30.00, SE_t0_ = 2.22, EM_t1_ = 30.42, SE_t0_ = 2.25; CON: EM_t0_ = 35.70, SE_t0_ = 2.62, M_t1_ = 33.00, SD_t0_ = 2.96). Yet, descriptive analysis of pre-/post-training performance differences at the individual level highlighted that EXP participants showed modulations ranging between −33% and +39% of their initial scores, while CON participants have shown modulations ranging between −18% and +3% of their initial scores.

**Figure 1:**
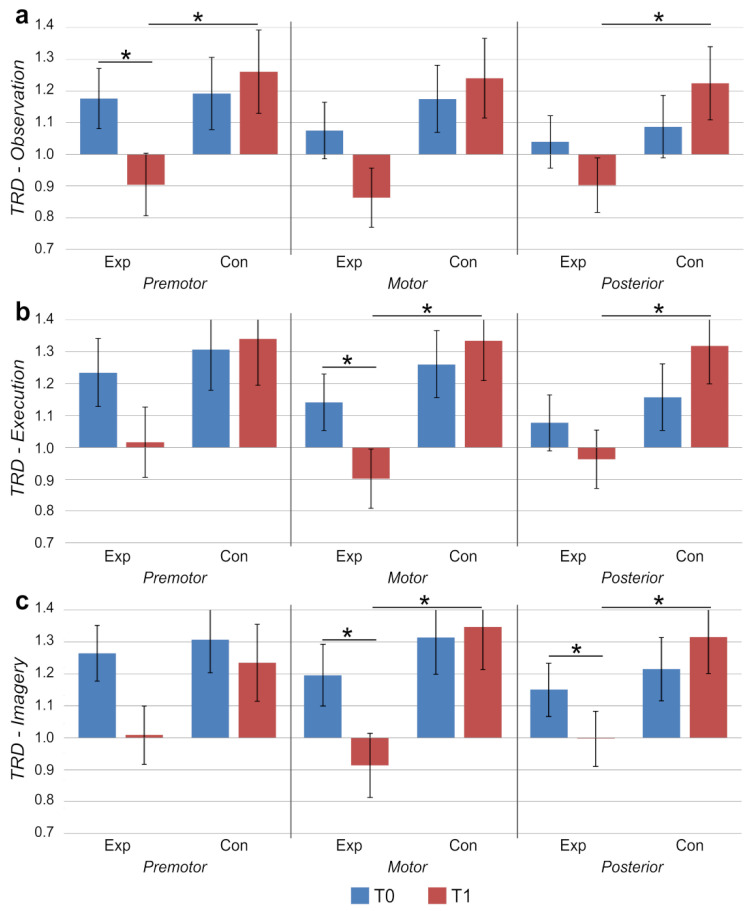
Task-related desynchronization (TRD) of the mu EEG band in correspondence to premotor, motor and posterior sites for the experimental (Exp) and control (Con) groups during: (**a**) action observation, (**b**) action execution, and (**c**) action imagery. T0: pre-training, T1: post-training. Bars represent group average ±1 SE. Stars mark statistically significant pair-wise comparisons.

The analysis of TRD of the mu band during OBS highlighted a significant interaction effect between Group and Time factors for the premotor region (Fz: *F*(1,69.37) = 5.869, *p* = 0.018) and the posterior region (Pz: *F*(1,71.37) = 4.524, *p* = 0.037). Pair-wise comparisons revealed that the EXP group showed greater mu TRD at T1 than at T0 in Fz (EM_t0_ = 1.18, SE_t0_ = 0.09, EM_t1_ = 0.91, SE_t0_ = 0.10; *d* = 0.50), and that the EXP group presented significantly greater mu TRD with respect to the CON group in correspondence to Fz and Pz at T1 (Fz: EM_EXP_ = 0.91, SE_EXP_ = 0.10, EM_CON_ = 1.26, SE_CON_ = 0.13; *d* = 0.78; Pz: EM_EXP_ = 0.90, SE_EXP_ = 0.09, EM_CON_ = 1.23, SE_CON_ = 0.12; *d* = 0.81; see Figure 1a).

Statistical models for TRD of the mu band during EXE highlighted a significant interaction effect between Group and Time factors for the motor region (Cz: *F*(1,73.21) = 4.570, *p* = 0.036) and the posterior region (Pz: *F*(1,68.05) = 5.141, *p* = 0.027). Pair-wise comparisons revealed that the EXP group showed greater mu TRD at T1 than at T0 in Cz (EM_t0_ = 1.14, SE_t0_ = 0.09, EM_t1_ = 0.90, SE_t0_ = 0.09; *d* = 0.41), and that the EXP group presented significantly greater mu TRD with respect to the CON group in correspondence to Cz and Pz at T1 (Cz: EM_EXP_ = 0.90, SE_EXP_ = 0.09, EM_CON_ = 1.33, SE_CON_ = 0.13; *d* = 0.96; Pz: EM_EXP_ = 0.96, SE_EXP_ = 0.09, EM_CON_ = 1.32, SE_CON_ = 0.12; *d* = 0.82; see Figure 1b).

The analysis of TRD of the mu band during IMM highlighted a significant interaction effect between Group and Time factors for the motor region (Cz: *F*(1,68.79) = 4.774, *p* = 0.032) and the posterior region (Pz: *F*(1,68.63) = 4.398, *p* = 0.040). Pair-wise comparisons revealed that the EXP group showed greater mu TRD at T1 than at T0 in Cz (EM_t0_ = 1.20, SE_t0_ = 0.10, EM_t1_ = 0.91, SE_t0_ = 0.10; *d* = 0.57) and in Pz (EM_t0_ = 1.15, SE_t0_ = 0.08, EM_t1_ = 1.00, SE_t0_ = 0.09; *d* = 0.52). Also, the EXP group presented significantly greater mu TRD with respect to the CON group in correspondence to Cz and Pz at T1 (Cz: EM_EXP_ = 0.91, SE_EXP_ = 0.10, EM_CON_ = 1.35, SE_CON_ = 0.13; *d* = 0.91; Pz: EM_EXP_ = 1.00, SE_EXP_ = 0.09, EM_CON_ = 1.32, SE_CON_ = 0.11; *d* = 0.78; see Figure 1c).

## 4. Discussion

The present study aimed at identifying and analyzing neurofunctional outcomes of somatic–perceptual learning mechanisms thought to be at the core of the Feldenkrais Method, a quite established embodied awareness practice whose application has extended, over the years, beyond physiotherapy and motor rehabilitation. Age and gender-matched participants underwent weekly sessions of standardized FM practice (namely ATM group sessions) or were put on a waitlist. The effects of the FM protocol on the complexity and integrity of the body structural map and on acuity and responsivity of the sensorimotor network to observation, execution, and imagination of complex gestures were assessed by comparing pre- and post-training psychometric and electrophysiological data.

Data analysis highlighted four main findings. Firstly, the representation of the body map showed no systematic modulation at the end of the FM protocol in the EXP group. Secondly, following the FM protocol, the pre-central scalp region was connoted by a significant increase of task-related desynchronization of the mu band during the action observation task. Thirdly, the central scalp region, at the end of the FM intervention, was connoted by a significant increase of mu task-related desynchronization during the action execution and imagery tasks. Fourthly, at the end of the FM protocol, the posterior regions of the sensorimotor network showed a significant systematic increase of mu task-related desynchronization across all the action-related tasks (i.e., action observation, execution, and imagery).

The hypothesis concerning the improvement of body structural map at the end of the FM protocol was, therefore, not verified. Feldenkrais himself [33] had originally stressed the importance of a definite representation of the body as a core precursor of efficient movement and behavior in humans and a core element of self-awareness, and suggested that working on somatic sensitivity and self-perception while moving helps in building a better and stronger awareness of the self, besides improving movement efficiency and sensorimotor acuity. While clinical reports and narrated examples from clinical practice often point out an increase in the quality and complexity of perceptual and affective representations of the body following regular FM practice, available empirical investigations only provide faint evidence in favor of such an expected outcome. Indeed, to our best knowledge, a modulation of perceived body-image is cited in just two studies on the method, it is mirrored by an unspecific improvement of the self-reported perception of body dynamics in acute patients after myocardial infarction [27] or by increased contentment with regard to problematic body zones and increased familiarity with their body in patients with eating disorders [28]. While such evidence hints at valuable implications for practice, it should be acknowledged that they only scrape the surface of the underlying construct and that the impact of FM practices on the development or improvement of body image and, in particular, of visual-spatial representations of the body should be object of further testing. The here-reported lack of systematic effects of the protocol on the complexity and integrity of the body structural map (as measured via an adapted and quantifiable version of the human figure drawing test) could be explained by the remarkable variability of participants’ performances at the test. Such variability might, indeed, have prevented inferential tests from pointing out statistically significant differences following the training period. Yet, qualitatively, it is worth reporting that the observed range of improvement in the experimental group with respect to the control group is higher and that about 60% of EXP participants showed a more complex representation of the body map after the protocol, findings that might be worth further investigation.

We acknowledge that the reported lack of statistically significant differences at the HFD test might be alternatively (or complementarily) explained by the intrinsic limitation of such a test. Indeed, while it allows for quantifying the complexity of the examinees’ body structural map and while its relatively restricted task instructions allow to reliably elicit the examinee’s inner visual-spatial representation of the body, such tests cannot be considered an exhaustive measure of representational levels constituting body image. As an example, the standardized version of the HFD that has been used in this study does not provide information on the conceptual or semantic connotations of the body and its parts (i.e., body semantics) nor on perceived levels of movement efficiency or functionality of the body and its parts. Complementing the HFD with other tools able to capture such features of the body representation might help to better investigate the effects of FM practice on body image and body schema.

Moving to electrophysiological findings, the analysis of the EEG mu rhythm allowed gathering of information on the degree of activation of sensorimotor structures and its modulation following the FM protocol. The amplitude of such a rhythm is, indeed, sensitive to action-related processes [22,23,24,34]. Namely, it has been suggested that desynchronization of mu oscillations recorded from scalp sites that overlay the sensorimotor regions is an index of sensorimotor cortical activation [35,36,37]. Cortical sources of the Mu oscillations were primarily observed as correspondence to central and parietal cortical regions [38,39], consistent with neuroimaging findings on the shared cortical substrate of action observation, execution, and imagery [40,41,42].

Notably, the fact that the modulation of cortical activation during observation of complex gestures in experimental participants was mainly observed in correspondence to the anterior and parietal locations is consistent with previous evidence concerning the contribution of frontal premotor and superior parietal structures to the human ability to perceive and code actions [23,41] and the topography of mu desynchronization during observation of movements [25]. Indeed, frontal structures play a crucial role in planning, programming, and encoding of motor acts [43,44,45], and superior frontal structures—i.e., dorsal premotor cortices—together with the posterior parietal cortex proved to code and process action-related information, well beyond the canonically-defined components of the mirror system [41]. Interestingly, it has been suggested that premotor areas could also be essential in the acquisition of effective motor awareness [46].

Again, a significant increase in cortical activation during both execution and imagination of complex motor acts was observed in correspondence of central and parietal scalp sites. Such finding is totally consistent with both the electrophysiological literature on functional and topographical features of the mu rhythm [22,23,24,34] and neuroimaging and electrophysiological literature on the common neurofunctional substrate for action execution and motor imagery [40,41,42]. Motor cortices, in particular, are now known to support even cognitive processes linked to movement, like preparation and conscious representation of an action, showing anticipatory activity for relevant movement sequences [47,48] and being modulated by attentional processes [49]. Therefore, the modulation of cortical activation in central scalp sites might mirror, given that they overlie motor regions, a modulation of individual ability of conscious programming and implementation of motor acts. Given the commitment, in FM practice, to conscious exploration and perfecting every single component of the motor process in order to achieve greater movement functionality, this peculiar outcome might actually be a marker of the effects of the learning process activated by the practice.

Finally, posterior cortical sites, where a modulation of mu desynchronization has been systematically observed across all action-related tasks, overlie somatosensory, associative, and posterior parietal areas. The integrated activation of the posterior parietal cortex, premotor cortex, and cerebellum allows movement planning and prediction of results [50]. Furthermore, parietal cortices play a crucial role in the integration of motor and perceptual information for the prediction of the sensory consequences of a movement, in the formation of multisensory representations of the body, and in the attribution of actions to oneself [51]. Consistently, lesions of parietal cortices—as well as disconnection of or direct damage to other nodes of the complex system encompassing the premotor loop, the limbic system, and the ventral attentional network [52]—can cause disorders in body and motor awareness, such as a distorted perception of the body or of its components, alteration of the body schema, anosognosia for hemiplegia, and disorders of agency attribution [53]. In addition, parietal areas, together with premotor ones, are involved in the perception and conscious representation of illusory movements following muscle-tendon vibration [54,55,56]. Taken together, such evidence outlines the specialization of parietal structures in creating a bridge between perceptual and motor information by integrating them to allow for the implementation of goal-directed actions and in shaping adaptive body representations [54,57]. The fact that mu rhythm showed a systematic modulation during each action-related task in participants who completed the FM protocol suggests that one of the first latent mechanisms to be affected by the practice might have to do with the integration of sensorimotor information flow to create situated representations of the body in action, which represent a common prerequisite to perceive and code observed actions and to plan and implement actual or imagined actions. The fact that mu desynchronization occurred over central and parietal locations during both action execution and imagination depose in favor of the potential of FM practice to modify processes subserving aware action programming.

## 5. Conclusions

To sum up, present findings add to the limited empirical literature on the effects of FM practice, with a specific focus on neurofunctional correlates of sensorimotor acuity. The desynchronization of the mu EEG band was used as a marker of cortical activation to explore the modulation of activation and responsivity of sensorimotor structures induced by regular practice with group ATM sessions. Such electrophysiological marker proved to be able to highlight consistent outcomes of practice, as well as to provide valuable information on potential mechanisms underlying the somatic-motor learning process thought to be at the core of the practice.

In addition, the above-presented findings hint at the potential of FM for empowering the responsivity of the sensorimotor network not only during motor execution (a primary target given the re-educational nature of the method), but also when complex gestures are observed and imagined. While such consistency could be traced back to the complementarity of neurophysiological processes underlying those action-related functions and to the well-known overlap of the neurofunctional substrates subserving them, it also hints at the pervasivity of the effects of FM practice. Furthermore, we believe that it points out the synergic effect of training awareness of own body in action, consistent with models that suggest that the consciousness of our movements is not an abstract mental process, separate from acting, but an intrinsic element the action implementation mechanisms supported by the extended frontoparietal sensorimotor network [58].

The present study is, however, not exempt from limitations. Firstly, the sample size is limited and drop-outs further lowered it. The strength of observed findings and their interpretation would therefore benefit from replication with additional samples, and subsequently from further testing with peculiar population groups connoted by suboptimal and/or optimized sensorimotor efficiencies, such as people with motor disturbances or athletes and performance artists, whose profession has likely led them to develop peculiarly optimized body representation and motor schemas. In addition, we acknowledge that the experimental design connoting the present study (i.e., randomized waitlist controlled design)—even though allowing to test the specific effects of FM practice on the complexity of body maps and on responsivity of sensorimotor structures during motor-related tasks, and allowing to preserve ethical principles of equal access to potential opportunities for empowerment and well-being—presents intrinsic limitations when it comes to comparative efficacy with respect to others treatments. Building on preliminary systematic studies on the effects of FM practice and on the mechanisms that foster those effects, future investigations should focus even on exploring the comparative advantages and shortcomings of such embodied awareness practice with respect to other alternative empowerment protocols. Furthermore, future research might include even field measurements of motor performance changes in order to better sketch the extent and practical implication of an empowerment protocol based on embodied awareness practices.

## Figures and Tables

**Table 1:** Demographic data for the whole sample and for experimental and control groups, and significance of their between-group statistical comparisons.

	EXP Group	CON Group	Total Sample	Sig.
Gender—M/F	5/9	3/8	8/17	n.s.
Age—Mean (SD)	49.93 (11.53)	52.27 (9.96)	50.96 (10.71)	n.s.

n.s., not significant.

## Data Availability

The data presented in this study are available from the corresponding author upon reasonable request.
